# Social Networks and Loneliness in the Blackfeet American Indian Community

**DOI:** 10.1007/s12529-025-10347-0

**Published:** 2025-01-30

**Authors:** Neha A. John-Henderson, Betty Henderson-Matthews, Zachary J. Wood, Skye Gilham, George Heavy Runner, Lester R. Johnson, III, Mary Ellen Lafromboise, Melveena Malatare, Emily M. Salois

**Affiliations:** 1 Department of Psychology, Montana State University, Bozeman, MT USA; 2 Center for American Indian and Rural Health Equity, Montana State University, Bozeman, MT USA; 3 Math and Science Department, Blackfeet Community College, Browning, MT USA; 4 Blackfeet Community, Bozeman, MT USA

**Keywords:** Loneliness, Social network size, Social integration, American Indian adults

## Abstract

**Background:**

While characteristics of an individual’s social network and reported loneliness may be linked, they can be distinct. Prior work indicates that gender moderates the relationship between social networks and loneliness; however, these relationships have not been investigated in American Indian adults. The current work investigates whether the relationship between characteristics of one’s social network (i.e., social network size and social integration) and loneliness is moderated by gender in a sample of Blackfeet American Indian adults.

**Method:**

At Wave 1 of a longitudinal research project, we used linear regression to test whether gender moderates the relationship between social network characteristics and loneliness in a sample of 275 Blackfeet American Indian adults living in the Blackfeet nation in Montana. Our analyses controlled for age, education, and symptoms and depression and anxiety.

**Results:**

Gender moderated the relationship between social network size and loneliness (*β* = − 0.15,* t*(265) = − 2.71, *p* = 0.01, *r*^2^ change = .04), and the relationship between social integration and loneliness (*β* = − 0.14, *t*(265) = − 2.68, *p* = 0.01, *r*^2^ change = .03). Women with small social networks reported significantly greater loneliness compared to men with similarly small social networks, and for women higher social integration (i.e., more social roles) related to lower loneliness, but this was not the case for men.

**Conclusion:**

Social network characteristics predict loneliness for Blackfeet women but not Blackfeet men in this sample. Future work should elucidate predictors of loneliness for Blackfeet men and consider whether daily changes in social connectedness predict changes in loneliness and whether changes in social networks predict changes in loneliness.

## Introduction

A social network refers to a group of people who are connected to each other through interactions and social relationships, and it is well established that one’s social network can influence health. In seminal work, over a 9-year period, adults with fewer social connections were significantly more likely to have died [1]. This relationship between social ties and mortality was independent of initial health status, smoking, socioeconomic status, obesity, and levels of physical activity. In subsequent years, researchers have continued to elucidate the relationship between social networks and health, with growing consideration of additional covariates and confounders [2].

There are many features of one social network that have been previously connected to health [2, 3]. For example, social network size, or the number of individuals in one’s social network, has been linked to numerous health outcomes. In one study, larger social network size was found to be related to lower all-cause mortality risk in older adults with diabetes [4]. Separately, smaller social networks related to higher risk for CVD [5]. Another feature of social networks linked to health is social integration. If an individual has a high level of social integration, then they have a large number of individuals in their network with whom they have frequent contact. High levels of social integration relate to optimal mental health outcomes across racial and ethnic groups [6].

Individuals with high social integration have many opportunities to receive and provide social support, and diverse social relationships can also promote positive health behaviors [7]. Prior work across racial and ethnic groups provides evidence of a relationship between characteristics of one’s social network (i.e., social network size and social integration) and a range of outcomes related to overall health [8–12].

Loneliness, or the discomfort one feels when they perceive a discrepancy between their desired social connection and their experience of social connection, is also related to poor mental health across racial and ethnic groups [13]. While loneliness and social network characteristics can be related [14], they may also be distinct. For example, in one previous study, loneliness and objective social disconnectedness were independently predictive of probability of having major depression disorder (MDD) and generalized anxiety disorder in a sample of older adults [8]. Prior research indicates that demographic factors including age, education level, and marital status are important predictors of loneliness, and importantly, these relationships are consistent across women and men [15–17].

### Social Networks and Loneliness in American Indians

Research on the relationship between social networks and loneliness in American Indians is relatively limited compared to the literature in other racial and ethnic groups. However, more research in this area is warranted given that loneliness is a known correlate of health [18–20], and given disproportionately high incidence of chronic health conditions in this population [21, 22].

Moreover, accumulating evidence highlights the relevance of social networks and loneliness for health-relevant outcomes in American Indian samples. In one sample of American Indian/Alaska Native adults, the size of one’s social network and their level of social integration were linked to perceptions of overall health. Specifically, smaller networks and lower social integration were related to poor perceived health for American Indian adults. Moreover, the relationship between social networks and perceptions of health was stronger for American Indian adults compared to non-Hispanic White adults in the sample [23]. Separately, in a sample of American Indian and Alaska Native emerging adults, social network characteristics related to symptoms of depression and anxiety and substance use [24, 25] Both studies utilized a sample of urban American Indian/Alaska Natives, so it is unknown if similar relationships would be observed in rural areas or on tribal reservations.

In a similar manner, while the precursors of loneliness and its implications for health have been widely studied across other racial and ethnic groups, to our knowledge, only one study has looked at the construct of loneliness in a sample of exclusively American Indian adults residing in the Blackfeet Nation. This study found that loneliness was related to sleep outcomes as measured by actigraphy [26]. It is possible that social networks may ultimately inform health in American Indian adults by shaping or contributing to perceived loneliness, however to date, this has not been investigated.

### Gender Differences in the Relationship Between Social Networks and Loneliness

Extant work across racial and ethnic groups indicates that the predictors of loneliness may vary as a function of gender. An Australian longitudinal focused on predictors of loneliness highlighted the unique antecedents of loneliness in men compared to women [27]. Separately, in a study of older adults, social contacts predicted loneliness for men, while depression, mobility problems, and widowhood predicted loneliness in women [28]. In Korean adults, a social network of family ties and marriage were related to loneliness in men, but not in women [29]. Finally, in a study of loneliness around the globe, differences in loneliness were observed between men and women [30]. Together, these findings indicate that the relationship between social network characteristics and loneliness may vary as a function of gender in American Indian adults, though to date, this remains unknown.

### Investigating the Relationship Between Gender, Social Networks, and Loneliness in Community Members of the Blackfeet Nation

The Blackfeet Nation, located in Northwest Montana, is bordered by Canada to the North and Glacier national Park to the West. A treaty with the United States Government in 1855 established federal recognition for the Blackfeet people. Following several agreements and executive orders, the Blackfeet aboriginal territory was reduced to its current size of 1.5 million acres in Northwest Montana. Enrolled members and their descendants reside on the remaining territories and adjacent communities, which now make up what is referred to as the Blackfeet Nation.

According to data from the 2022 United States census, there are 4.4 people per square mile in the Blackfeet Nation, compared to an average of 93.29 people per square mile overall in the United States, and an average of 8 people per square mile in the state of Montana [31]. Given the very low population density in the Blackfeet Nation, social networks may be a particularly important informant of loneliness for community members. For example, even though an individual may feel physically isolated from others in the community because of low population density, they may have a large social network allowing for regular contact by phone or otherwise providing for higher levels of social interaction and integration. Furthermore, the employment rate in the Blackfeet Nation is 11.3% lower than the employment rate in the United States, the average annual income is about half the average amount in the United States, and 30.9% of community members are below the federal poverty line (more than twice the amount in the United States) [31].

Prior work examining roles and expectations of women in two American Indian tribes found that women are viewed as centers of family, and prioritize family over economic aspirations [32]. This suggests that correlates of social networks and implications of social networks for loneliness may differ for Blackfeet women compared to Blackfeet men. Investigating the relationship between social networks and loneliness in the Blackfeet Community provides a unique opportunity to understand these constructs and their relation to each other, and to investigate whether gender moderates their relationship in the context of a rural tribal reservation and in the context of financial and economic hardships present in the Blackfeet Nation.

The current investigation builds upon known links between social networks, loneliness, and a host of health-relevant outcomes [2, 3, 13], on our prior work highlighting the importance of social connectedness in the context of health for Blackfeet adults [26, 33], and on prior work indicating gender differences in correlates of social networks and loneliness [27–29]. Specifically, we investigate whether the relationship between social network characteristics and reported loneliness is moderated by gender in a sample of Blackfeet American Indian adults residing in the Blackfeet Nation in Northwest Montana. Based on prior work indicating the prioritization of social relationships for American Indian Women [32], we hypothesized that the relationship between social networks and loneliness would be evident for Blackfeet American Indian women, but not for Blackfeet American Indian men. Given that marital status is also related to loneliness [16, 34], we investigated whether marital status interacted with gender, and social network characteristics to predict loneliness, with the hypothesis that the relationship between social networks and loneliness in women may only be evident in those who are not married.

## Methods

The current project is founded upon a long-standing partnership between a Psychology Professor at Montana State University and Blackfeet Community members and faculty and students at Blackfeet Community College. This partnership and program of work is itself built upon many years of collaborative research between Blackfeet Community College and Research faculty at Montana State University. The current partnership is informed by Community Based Participatory Research principles which emphasize the importance of an equitable partnership between researchers and community members [35]. As such, a community advisory board comprised of Blackfeet Community members reviewed and helped to select and design the research questions, study design, selected measures, and guided data interpretation.

Based on many years of pilot studies and research investigations [36], the research team developed the current study “*Aa Koo Moo Waap*,” which can be translated to “People Coming Together” in Blackfeet. The primary goal of *Aa Koo Moo Waa*p is to elucidate the relationship between social connectedness and health in the Blackfeet Community over a period of 2 years. The current manuscript is based on data from Wave 1 of *Aa Koo Moo Waap.* The research team recruited 280 Blackfeet Community members aged 18–65 to participate in the study.

Eligibility criteria included being between the ages of 18 and 65, self-identification as American Indian, and current residence in the Blackfeet Nation. Prior to the start of data collection, eligibility was confirmed using a survey on the Qualtrics platform for most participants. For other participants who did not have access to email or the internet, we confirmed eligibility over the phone or using an in-person interview. Once eligibility was confirmed, participants were scheduled to come to the Blackfeet Community College for data collection. The final sample was comprised of 275 American Indian adults residing in the Blackfeet nation between the ages of 18 and 65. All participants completed a survey using the Qualtrics platform. The survey included multiple measures of social connectedness, mental health, physical health, sleep health, trauma (recent and childhood), civic engagement, perceived discrimination, and other psychosocial variables.

### Measures

#### Demographics

Participants self-reported their age, gender, marital status, and highest level of education. Education was coded using the following categories: 1 = did not complete high school, 2 = high school diploma or GED, 3 = some college but no degree, 4 = Associate degree, 5 = Bachelor’s degree, 6 = Master’s degree or higher. Gender was coded as male = 0 and female = 1 and marital status was coded as unmarried = 0 and married = 1. We used the Hospital Anxiety and Depression Scale (HADS) [37] to measure current symptoms of depression and anxiety. The HADS consists of a seven-item subscale to measure symptoms of anxiety and a seven-item subscale to measure symptoms of depression. The scale for each item was 0–3, and higher scores on each subscale reflect more symptoms. Age and education were used as covariates in our analyses based on known relationships between these variables and loneliness [30, 37, 38].

#### Social Network Index

We used the Social Network Index (SNI) as a measure of social integration and social network size [39]. The SNI measures social integration and the size of one’s social network. Social integration reflects the number of different types of high contact social roles in which an individual participates. For a social relationship to be categorized as high contact, the individual must indicate that they have contact with that person either in-person or on the phone at least once every 2 weeks. Twelve social relationships are included: spouse, parent, parent-in-law, child, child-in-law, close relative, close friend, church/religious group member, student, employee or coworker, neighbor, volunteer, or other group member. Separately, social network size is computed by summing the number of people with whom an individual has contact at least once every 2 weeks.

#### Loneliness

We used the short loneliness scale [40], as a measure of self-reported perceived loneliness. The measure has three items which use a 4-point rating scale to measure the frequency with which participants feel isolated, lacking in companionship, and left out. This measure of loneliness includes a subjective measure of social isolation, or the degree to which an individual *feels* socially isolated, which is distinct from more objective measures of social isolation such as social network size. In the current sample, the scale had good reliability (Cronbach alpha = 0.88).

## Results

Descriptive statistics for the sample are listed in Table 1 (*N* = 275) along with bivariate correlations. Gender was related to social network diversity and size, with females having larger and more diverse social networks. The highest level of education was also related to both social integration and size, with higher level of education relating to larger networks and more social integration.

**Table 1:** Descriptive Statistics and Bivariate Correlations (*N *= 275)

Variable	M	SD	1.	2.	3.	4.	5.	6.	7.	8.	9.
1. Age	38.67	14.30	-	.081	.080	.221**	.196**	.076	–.032	–.070	–.197**
2. Gender	64.4% Female			-	.117	–.079	.169**	.187**	–.111	–.023	.118
3. Education	31.8% High school diploma or GED				-	.015	.278**	.245**	–.032	–.131*	–.023
4. Marital Status	29.5% married					-	.151*	.116	–.124*	–.065	–.071
5. Social network size	18.29	8.02					-	.743**	–.157**	–.363**	–.217**
6. Social integration	5.44	2.09						-	–.093	–.279**	–.101
7. Loneliness	5.89	2.42							-	.448**	.449**
8. Symptoms of Depression	6.03	3.43								-	.653**
9. Symptoms of Anxiety	8.90	4.37									-

Gender coded as 1 = male, 2 = female; Marital status coded as 0 = not married, 1 = married

^*^p < 0.05, **p < 0.001

### Social Networks and Loneliness

**Fig. 1:**
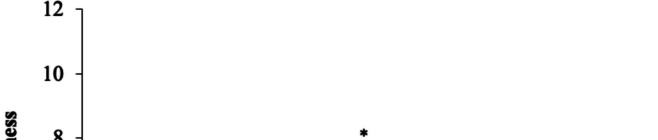
Results from hierarchical regression with gender and social network size predicting loneliness. This regression includes the covariates of age, highest level of education, and current symptoms of depression and anxiety

We investigated whether the relationship between qualities of social networks and perceived loneliness was moderated by gender. More specifically, we tested our hypothesis that social network characteristics (i.e., social network size and social integration) would inform loneliness for Blackfeet women, but not for Blackfeet men. We wanted to establish that observed relationships were independent of the contribution of factors that are known predictors of loneliness, so we included the previously described covariates in our overall model. In a hierarchical linear regression, we entered age and highest level of education in step 1 as covariates, gender and social network size in step 2, and the interaction between gender and social network size in step 3. The interaction between gender and social network size was a statistically significant predictor of perceived loneliness (*β* = − 0.15, *t*(265) = − 2.71,* p* = 0.02, *r*^2^ change = 0.04). Figure 1 displays the pattern of this interaction and Table 2 presents the full results of this regression model. To probe this interaction, we conducted simple slope analyses. For these analyses, “large social networks” refers to 1 SD above the mean in social network size and “small social networks” refers to 1 SD below the mean in social network size. For men in this sample, there was no statistically significant difference in loneliness between those who had large social networks and those who had small social networks (*b* = − 0.06, *t*(265) = − 0.23, *p* = 0.81), while for women in this sample, those who had large social networks reported significantly lower loneliness compared to those women who had small social networks (*b* = − 0.86, *t*(265) = − 3.77, *p* < 0.001). In comparing men and women in this sample with small social networks, women had significantly higher levels of loneliness compared to men (*b* = 0.68, *t*(265) = 2.61,* p* = 0.01).

**Table 2:** Hierarchical linear regression with social integration and gender predicting loneliness. Gender coded as male = 0, female = 1. *CI*, confidence interval

Step	Variable	*B*	95% CI [LL, UL]	*β*	*t*	*p*	*R*^2^ change for step
1	Age	0.05	[− 0.13, 0.02]	0.03	0.54	0.59	
	Education	0.14	[− 0.05, 0.32]	0.08	1.46	0.15	
	Anxiety symptoms	0.15	[0.07, 0.23]	0.28	3.86	< 0.001	
	Depressive symptoms	0.21	[0.11, 0.31]	0.28	4.00	< 0.001	
2	Age	0.01	[− 0.13, 0.02]	0.31	0.55	0.58	
	Education	0.14	[− 0.06, 0.33]	0.08	1.38	0.17	
	Anxiety symptoms	0.14	[0.06, 0.22]	0.26	3.58	< 0.001	
	Depressive symptoms	0.20	[0.96, 0.31]	0.28	3.76	< 0.001	
	Gender	0.19	[− 0.71, 0.45]	0.08	1.43	0.15	
	Social network size	− 0.12	[− 0.41, 0.17]	− 0.05	− 0.82	0.41	
3	Age	0.00	[− 0.02, 0.02]	0.02	0.34	0.73	
	Education	0.17	[− 0.02, 0.37]	0.10	1.72	0.09	
	Anxiety symptoms	0.13	[0.06, 0.21]	0.24	3.35	< 0.001	
	Depressive symptoms	0.20	[0.10, 0.31]	0.28	3.81	< 0.001	
	Gender	0.25	[− 0.01, 0.51]	0.11	1.89	0.06	
	Social network size	− 0.15	[− 0.44, 0.14]	− 0.06	− 1.01	0.32	
	Gender × Social network size	− 0.31	[− 0.54, − 0.09]	− 0.15	− 2.71	0.01	.04

**Fig. 2:**
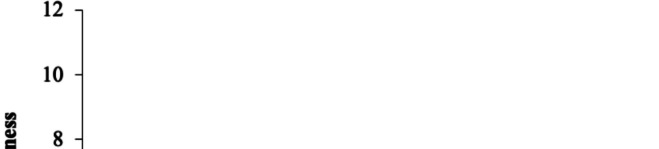
Results from hierarchical regression with gender and social integration predicting loneliness. This regression includes the covariates of age, highest level of education, and current symptoms of depression and anxiety

In a separate linear regression, we entered the previously listed covariates in step 1, gender and social integration in step 2, and the interaction between gender and social integration in step 3. The interaction between gender and social integration was a statistically significant predictor of perceived loneliness (*β* = − 0.14, *t*(265) = − 2.68, *p* = 0.01, *r*^2^ change = 0.03). Figure 2 displays the pattern of this interaction and Table 3 presents the full results of this regression model. To further probe this interaction, we conducted simple slope analyses. For these analyses, “high social integration” refers to 1 SD above the mean in social integration, and “low social integration” refers to 1 SD below the mean in social integration. Women with low social integration had significantly higher levels of reported loneliness compared to men with similarly low social network integration (*b* = 0.60,* t*(265) = 2.30,* p* = 0.02) and women who had high social integration (1 SD above the mean) had significantly lower levels of reported loneliness compared to women who had low social integration (*b* = − 0.56,* t*(265) = − 2.46, *p* = 0.01).

**Table 3:** Hierarchical linear regression with social integration and gender predicting loneliness. Gender coded as male = 0, female = 1. *CI*, confidence interval

Step	Variable	B	95% CI [LL, UL]	*β*	*t*	*p*	*R*^2^ change for step
1	Age	0.01	[− 0.01, 0.02]	0.03	0.54	0.59	
	Education	0.14	[− 0.05, 0.32]	0.08	1.46	0.15	
	Anxiety symptoms	0.15	[0.07, 0.23]	0.28	3.86	< 0.001	
	Depressive symptoms	0.21	[0.11, 0.31]	0.28	4.00	< 0.001	
2	Age	0.00	[− 0.11, 0.02]	0.03	0.45	0.65	
	Education	0.13	[− 0.06, 0.33]	0.08	1.33	0.19	
	Anxiety symptoms	0.14	[0.07, 0.22]	0.26	3.59	< 0.001	
	Depressive symptoms	0.21	[0.10, 0.31]	0.29	3.88	< 0.001	
	Gender	0.19	[− 0.07, 0.45]	0.08	1.41	0.16	
	Social integration	− 0.09	[− 0.37, 0.19]	− 0.04	− 0.61	0.55	
3	Age	0.00	[− 0.02, 0.02]	0.01	0.21	0.83	
	Education	0.17	[− 0.03, 0.36]	0.10	1.69	0.09	
	Anxiety symptoms	0.13	[0.05, 0.21]	0.24	3.32	0.001	
	Depressive symptoms	0.21	[0.11, 0.31]	0.29	3.98	< 0.001	
	Gender	0.22	[− 0.04, 0.48]	0.09	1.67	0.16	
	Social integration	− 0.10	[− 0.38, 0.18]	− 0.04	− 0.73	0.47	
	Gender × Social integration	− 0.29	[− 0.51, − 0.08]	− 0.14	− 2.68	0.01	.03

Finally, we tested whether marital status moderated the interaction between gender and social network characteristics to predict loneliness. In the first linear regression, we entered age and education in step 1 as covariates. In step 2, we entered marital status, gender, and social network size; in step 3, we entered the interaction between marital status and gender, the interaction between marital status and social network size, and the interaction between social network size and gender; and in step 4, we entered the three-way interaction term for marital status, gender, and social network size. This three-way interaction was not a statistically significant predictor of loneliness (*β* = 0.03,* t*(263) = 0.88, *p* = 0.38). Using the same approach, but replacing social network size with social integration, similar to the findings with social network size, the three-way interaction between marital status, gender, and social integration was not a statistically significant predictor of loneliness (*β* = 0.07,* t*(263) = 0.41, *p* = 0.68).

## Discussion

To our knowledge, the current investigation is the first to investigate the relationship between social networks and loneliness in a sample of exclusively American Indian adults residing on a tribal reservation. Based on prior evidence of gender-specific correlates of loneliness in other racial/ethnic groups [27–29], we investigated whether social network characteristics (i.e., size of social network and social integration) would be differentially related to loneliness for Blackfeet American Indian men compared to Blackfeet American Indian women. Our moderation analyses revealed that social integration was a significant predictor of self-reported loneliness for Blackfeet women, but not for Blackfeet men, in this sample. Specifically, Blackfeet women with greater social integration reported significantly lower levels of loneliness compared to women with lower social integration. We did not observe this relationship for Blackfeet men in this sample, suggesting that social integration may be more relevant in informing perceptions of loneliness for women in this sample compared to men.

In a similar manner, social network size did not predict differences in loneliness for Blackfeet men in this sample; however, it was a significant predictor of loneliness for women in this sample. When comparing Blackfeet men with small social network sizes to Blackfeet women with similarly small social network sizes, women in this sample had significantly higher levels of loneliness.

Overall, these findings suggest that in the current sample, both social network size and social integration inform perceptions of loneliness for Blackfeet women, but do not inform perceptions of loneliness for Blackfeet men. Given that social networks do not appear to be significant informants of loneliness for Blackfeet men in the current sample, future research should focus on identifying the relevant factors which either contribute to or protect from loneliness for this group. It is also possible that there are gender differences in willingness to report feelings of loneliness in the community.

We also considered whether the interaction between social network characteristic and gender in predicting loneliness would be further modified by marital status. Specifically, we tested the possibility that social network characteristics may only predict loneliness in women who were not married. Our analyses did not provide evidence to support this hypothesis, instead suggesting that social network characteristics are important informants of loneliness for Blackfeet women regardless of whether they are currently married or not.

The current findings should be interpreted in the context of the Blackfeet Nation and its community members. Specifically, as noted earlier, the Blackfeet Nation is characterized by a unique social, cultural, and economic context. First, distinct from prior work investigating these constructs in urban American Indians, the participants in the current sample reside on tribal territory or in immediately adjacent communities in rural Northwest Montana. The relatively low population density may affect the nature of social networks and the degree to which characteristics of social networks inform loneliness. The low population density may encourage more social interactions through phone, internet, or social media, and this should be investigated in future work.

Furthermore, the low employment rate and poverty present in the community may limit opportunities for social engagement, opportunities for social participation, and expanding one’s social network. The current findings provide support for our hypothesis that social networks would be a more relevant informant of loneliness for women compared to men. This hypothesis was rooted based on prior work in American Indian tribal communities, which found that women in these communities are viewed as the center of the family, prioritizing social relationships over economic concerns [32]. Our findings provide further support of the centrality and importance of social relationships and social roles for American Indian women. Based on these findings, interventions which work to expand the social networks and social roles for American Indian women living in the Blackfeet Nation could reduce loneliness.

Future research utilizing daily monitoring could examine whether the nature and frequency of daily social interactions inform loneliness in similar or distinct ways for men and women in the Blackfeet Community. This approach could also allow for investigation of the day-to-day changes in social connectedness and the stability of loneliness in the community. In other words, it will be important to understand how stable loneliness is for community members, and whether loneliness fluctuates in correspondence with daily changes in social interactions and other objective measures of social connectedness. The findings from the current work suggest that interventions which work to increase the size and diversity of Blackfeet women’s social network could reduce loneliness. By reducing loneliness, it is possible that such interventions could promote positive downstream mental and physical health outcomes given the known ties between loneliness and health.

An important limitation of the current work is its cross-sectional data, which does not allow for testing for directionality of effects. However, since *Aa Koo Moo Waap* is a 2-year longitudinal study, data collected in subsequent waves will allow for investigation of whether characteristics of Blackfeet adults’ social networks at the start of the study predict later loneliness, or whether initial loneliness predicts subsequent changes in social network size or diversity of social roles. The longitudinal study design will also let us test whether social networks and loneliness independently or cumulatively predict changes in many indices of physical and mental health.

Another limitation of the current data is the time of data collection. Data for Wave 1 of the *Aa-Koo-Moo-Waap* project was collected during the second two weekends of April 2023. Levels of social activities and social participation vary for community members with the change in seasons. During the summer months, there are many more social activities and opportunities for social engagement including the Rodeo, golf events, youth basketball games, and pow wows. Due to seasonal changes in the frequency of social events, social networks and perceived loneliness may also vary as a function of the seasons. It is possible that social networks may be the smallest and the least diverse in the winter months, and that in turn, loneliness is at its highest during these months.

Overall, the current work makes an important initial contribution to knowledge on the relationships between social networks and loneliness in Blackfeet American Indian adults. As noted previously, understanding predictors of loneliness is of great importance for this population, given known links between loneliness and many chronic health conditions which disproportionately affect members of the Blackfeet Community. Building upon the findings here and providing a more nuanced understanding of the pathways through which social networks and loneliness affect downstream health outcomes are essential to inform effective interventions designed to address enduring health inequities.

## Data Availability

While the authors have all primary data, the data belongs to the Blackfeet tribe. Requests to access data will be reviewed by the tribal council.
